# Trends in COVID-19 Publications: Streamlining Research Using NLP and LDA

**DOI:** 10.3389/fdgth.2021.686720

**Published:** 2021-07-06

**Authors:** Akash Gupta, Shrey Aeron, Anjali Agrawal, Himanshu Gupta

**Affiliations:** ^1^Department of Engineering, University of Cambridge, Cambridge, United Kingdom; ^2^Electrical Engineering and Computer Science, University of California, Berkeley, Berkeley, CA, United States; ^3^Harmony School of Innovation – Sugar Land (High School), Sugar Land, TX, United States; ^4^Valley Health System, Ridgewood, NJ, United States

**Keywords:** natural language processing, latent dirichlet allocation, COVID-19, trends, LitCovid, topic model, Pubmed

## Abstract

**Background:** Research publications related to the novel coronavirus disease COVID-19 are rapidly increasing. However, current online literature hubs, even with artificial intelligence, are limited in identifying the complexity of COVID-19 research topics. We developed a comprehensive Latent Dirichlet Allocation (LDA) model with 25 topics using natural language processing (NLP) techniques on PubMed® research articles about “COVID.” We propose a novel methodology to develop and visualise temporal trends, and improve existing online literature hubs.

Our results for temporal evolution demonstrate interesting trends, for example, the prominence of “Mental Health” and “Socioeconomic Impact” increased, “Genome Sequence” decreased, and “Epidemiology” remained relatively constant. Applying our methodology to LitCovid, a literature hub from the National Center for Biotechnology Information, we improved the breadth and depth of research topics by subdividing their pre-existing categories. Our topic model demonstrates that research on “masks” and “Personal Protective Equipment (PPE)” is skewed toward clinical applications with a lack of population-based epidemiological research.

## Introduction

The COVID-19 outbreak was officially declared a pandemic by the World Health Organization in March 2020 (1). As the number of COVID-19 cases and deaths has increased, so has the research. Searching “COVID” in PubMed®'s database gives a list of over 32,000 unfiltered publications (as of July 2020). Due to the overwhelming stream of papers, there is now an urgent need for tools to automate the categorical organisation of research. More importantly, to sufficiently address COVID-19 and future pandemics, it is necessary to streamline the research and development process by allowing for quick identification of research areas that are either gaining popularity or lacking adequate research.

Latent Dirichlet Allocation (LDA) is an unsupervised topic modelling technique used to learn hidden topics within a corpus (2). It assumes topics are a soft clustering of words and outputs two probability distributions: a distribution of topics in the corpus, and distributions of words across each topic. Currently, LDA, with the aid of natural language processing (NLP) methodologies, has been used to investigate the response to government policies (3), analyse public sentiment on social media (4) and the news (5), and understand general research hotspots in publications (6) as well as global trends (7). Most of these topic models either use a small number of topics or group a large number of topics into overarching themes, which eases comprehension at first glance. However, for a more in-depth analysis, a better understanding of the complexity of topics within each theme is required, which cannot be captured by searching for that topic in a literature repository using a simple query.

The National Center for Biotechnology Information (NCBI) has developed LitCovid, a central repository for curated COVID-19 research (8). The hub uses a combination of human and machine-learning methods to provide publications categorised by eight topics alongside temporal and geographic distributions. Although very useful, the defined categories are overly broad, limiting rapid assimilation of the ongoing research.

Here, we describe and implement an NLP methodology using LDA to identify topics in COVID-19 research. We then visualise and evaluate temporal trends to recognise the dominant and under-represented areas of research. We also apply our methodology to the LitCovid dataset and further subdivide their categories into topics. Although our results focus on COVID-19, we believe our method is generalisable and sustainable for evaluating rapidly evolving research fields.

## Methods

### Data Sourcing

Our dataset is extracted using Entrez Programming Utilities (9) from the PubMed® Application Programming Interface (API) through the BioPython package. The API returns a list of metadata on all documents retrieved in a search query, which was the term “COVID.” For each abstract, the PubMed® Document ID (PMID), date of publication on PubMed®, and abstract are stored.

A similar process is repeated for LitCovid abstracts. LitCovid (8), aided by machine-learning methods, manually assigns the articles into the eight categories of “Mechanism,” “Transmission,” “Diagnosis,” “Treatment,” “Prevention,” “Case Report,” “Forecasting,” and “General.” We use the NCBI Coronavirus API to extract the PMIDs corresponding to abstracts in each LitCovid category. We perform the same data cleansing process on the LitCovid abstracts as with the PubMed® abstracts.

For significantly large corpus sizes with a variety of journals, abstracts produce very similar topics compared to those produced by using the full text (10). Hence, we decided to use abstracts (3) due to the further benefits of much-reduced computation and free accessibility, aiding in reproducibility. A small number of abstracts are excluded from the corpus (*N* = 99) for the following reasons: those with <50 characters (*N* = 73), and others with a sentence including “This corrects the article DOI […]” (*N* = 26).

### Overview of the LDA Model

LDA is a standard topic modelling algorithm that learns the hidden topic structure within a corpus (2). Each topic has a weight within the corpus and is represented by a set of related words. We used Gensim's Python implementation of LDA to develop a topic model using a bag-of-words representation of the pre-processed training set. Further details about the NLP methodology and optimisation process are explained in the Supplementary Material. In brief, we set an upper limit on the number of topics to 50 and optimised hyperparameters based on the coherence value. Additionally, we selected a random seed to use in the training process to ensure replicable results. We implemented the methodology in Python.

### Temporal Evolution of Topics

**Figure 1:**
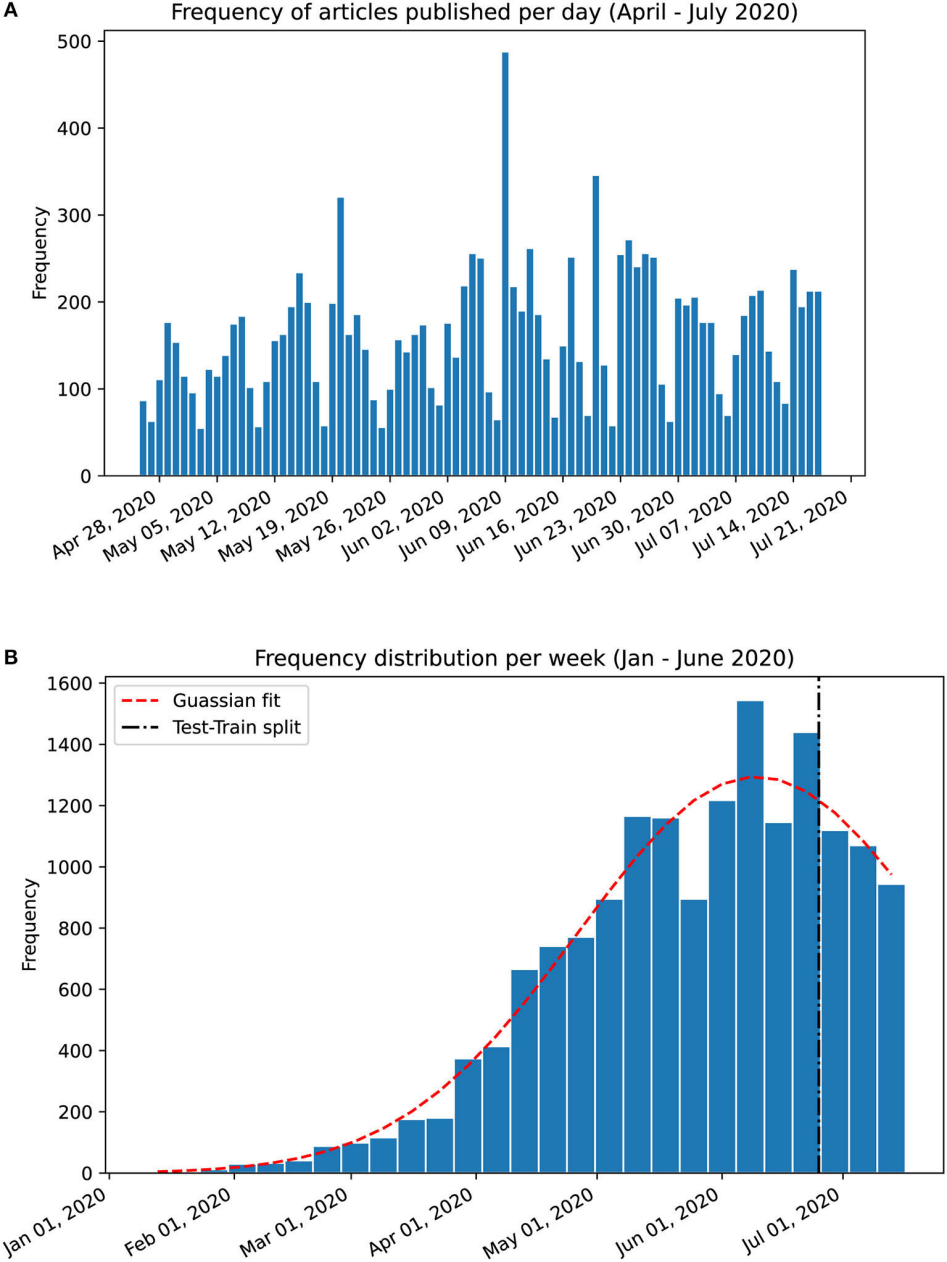
Frequency charts of abstracts published in PubMed®. **(A)** Per day frequency shows a weekly pattern. **(B)** Per week frequency from January 17th to July 17th 2020 with a gaussian curve fitting (shown in red dashed line). The training set was split from the testing set at the 81% mark on June 22nd 2020. The weekly release pattern informed our decision to analyse trends on a per-week basis.

To evaluate the temporal trends, we propose a novel method, which is applied to both PubMed® and LitCovid abstracts to produce an intuitive visualisation of the weekly temporal evolution of topic proportions. Analysis on a day-by-day basis can result in too much noise; instead, the documents are grouped by week, which is further justified by the apparent weekly release pattern of papers as shown in Figure 1A. However, since the weeks in January 2020 contain a substantially low number of papers, they are grouped into a single month. To evaluate how well the temporal evolution can predict future releases, we assign the weeks into training and testing sets with an approximate ratio of 8:2 (which is shown by the test-train split in Figure 1B).

For each week, in both the training and testing sets, all abstracts are combined into a single document. Applying the LDA model to these documents returns an estimate of the proportions of each topic that week. These results are graphed to produce a temporal topic evolution that is compared between PubMed® and LitCovid. We also create a heatmap to visualise all topics in the corpus in a concise chart.

### Subdivision of LitCovid Categories

For each of the eight LitCovid categories, all abstracts are combined into a single document. Applying our model to the documents returns the proportions of each LDA topic in each LitCovid category. Statistical analysis (as described in the next section) is used to evaluate the categories and subdivide them into corresponding LDA topics.

### Statistical Analysis

We incorporate tools from the Gensim Package (11) to perform statistical analyses on the topic model. We use perplexity (how surprised a model is to a sample) and topic difference to track the model convergence. The final performance of the model is evaluated using a coherence score (12).

Since LDA is a probabilistic model (2), the methodology used to capture temporal trends, as well as the subdivision of LitCovid categories is statistical. Applying the LDA topic model to a document returns a proportion for each topic, which is then used for further analysis.

Due to the non-linear nature of the temporal trends, a qualitative account of the trend fitting between the testing and training sets is discussed. Temporal trends of PubMed® and LitCovid publications are compared using Normalized Euclidean Distance, and topics are compared to each other using Jaccard Distance, a comparison between disjoint terms in two models (13).

LitCovid categories are compared against each other as well as the PubMed® corpus using Hellinger Distance (H):


$$H(P,Q)=\frac{1}{\sqrt{2}}\sqrt{\sum_{i=1}^{k}{\left(\sqrt{p_{i}}-\sqrt{q_{i}}\right)}^{2}}$$

Where k is the number of topics; P and Q are the topic proportions returned by applying our LDA model to each LitCovid category.

## Results

The PubMed® API returned 16,445 abstracts with the search query “COVID” (as of July 17th, 2020). We exclude 73 abstracts with <50 characters and 26 abstracts with extraneous information, resulting in 16,346 abstracts in the corpus from January 17th to July 17th, 2020. The distribution of these follows a gaussian curve with mean on June 9th, 2020 (Figure 1B). The corpus is split (81:19) as per section Temporal Evolution of Topics: 13,212 abstracts for training and 3,134 abstracts for testing. The cutoff date for the training set is June 22nd (inclusive).

**Figure 2:**
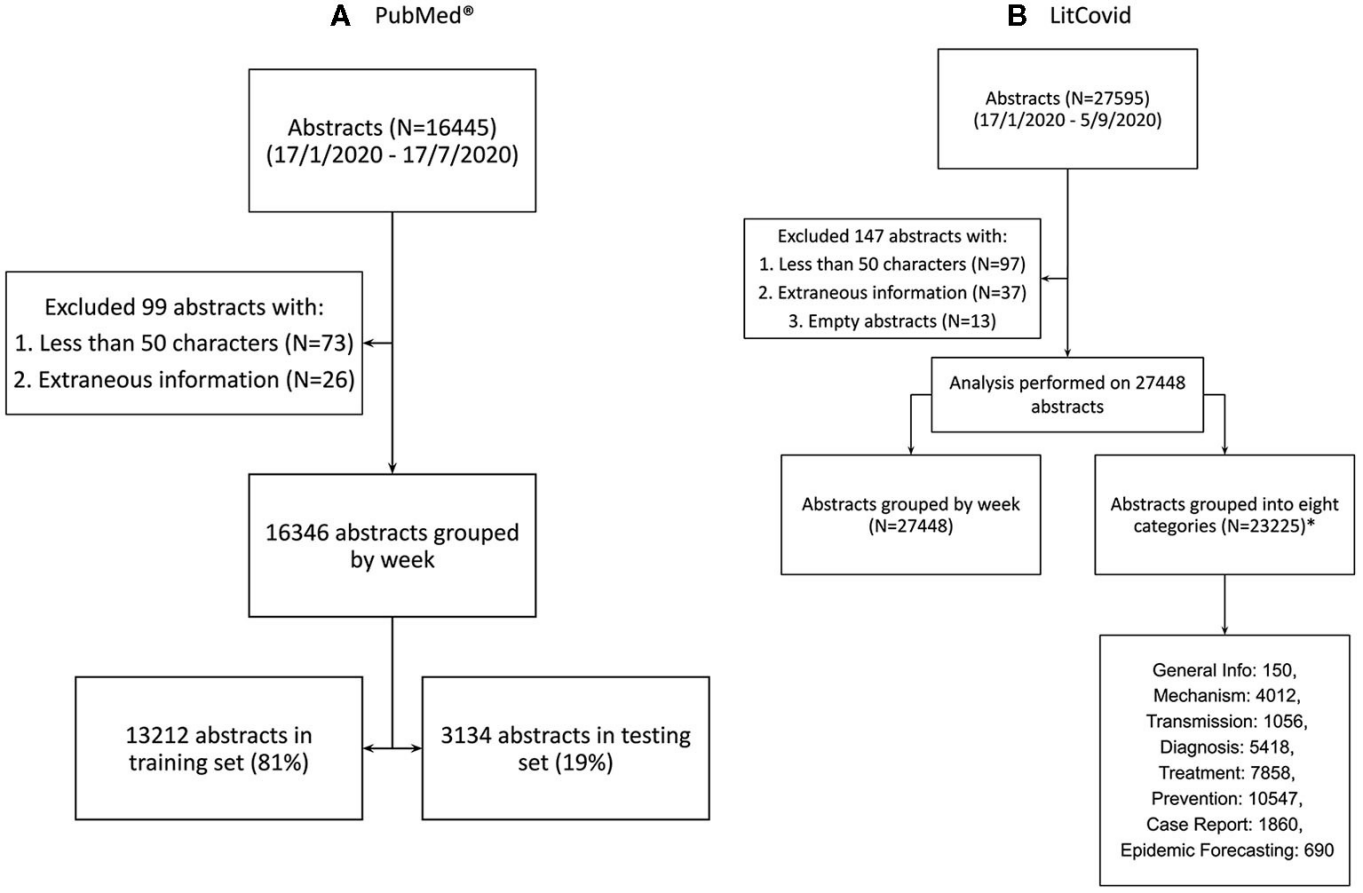
Data sourcing and splitting. **(A)** Abstracts from PubMed® using the search term “COVID” **(B)** Abstracts from LitCovid Dataset using the search term “coronavirus,” “ncov”, “cov,” “2019-nCoV,” “COVID-19,” and “SARS-CoV-2” (14). *Note that 4,223 LitCovid abstracts were not associated with any LitCovid category. Note that the total number of abstracts in all listed LitCovid categories is greater than the number of unique abstracts because an abstract can be placed in more than one category.

LitCovid generates 27,595 abstracts (as of September 8th, 2020) from the entirety of their dataset. We exclude 13 empty abstracts, 97 abstracts with <50 characters, and 37 abstracts with extraneous information. The final number of abstracts is 27,448, ranging from January 17th to September 5th 2020. 4,223 LitCovid abstracts could not be associated with any category and 23,225 are associated with at least one of the eight categories. The distribution of these categories is in Figure 2.

The LDA model is trained on 13,212 abstracts to produce a topic model with 25 topics. The final perplexity is 185.6, and the coherence score is 0.526. The average normalised Jaccard distance between the topics is 0.923 (STD 0.048) with a minimum of 0.701 between Topic 23 (“Gastroenterology”) and Topic 13 (“Paediatrics”). A list of all the topics is displayed in Table 1 alongside the top 10 relevant words and our summary interpretation based on the top words and abstracts associated with each topic. The topic of “Health care, telemedicine” has the highest proportion of 9.4%, whereas “pregnancy” has the lowest proportion of 1.0%.

**Table 1:** Topic description and summary about COVID-19 produced by LDA model.

**Topic**	**Top 10 Most Relevant Words**	**Topic summary**
1	Care, pandemic, service, telemedicine, health_care, need, resource, challenge, telehealth, healthcare	Health care, Telemedicine
2	Number, case, model, country, estimate, epidemic, estimated, data, daily, rate	Epidemiology
3	Case, day, chest, pneumonia, lesion, symptom, consolidation, fever, group, showed	Pulmonary
4	China, outbreak, epidemic, world_health, prevention_control, case, disease, novel_coronavirus, public_health, january	Disease Outbreak
5	Review, article, pandemic, research, vaccine, current, paper, scientific, literature, evidence	Scientific Development
6	Surgery, surgical, procedure, surgeon, hospital, dental, ppe, pandemic, emergency, staff	Surgery
7	Mortality, study, compared, included, outcome, risk_factor, higher, group, hospitalized, severe	Clinical Outcomes
8	Participant, survey, anxiety, questionnaire, mental_health, respondent, psychological, stress, fear, perceived	Mental Health
9	Disease, cardiovascular, acute_respiratory, cardiac, distress_syndrome, severe, ards, cytokine_storm, syndrome, cardiovascular_disease	Cardiovascular
10	Treatment, trial, clinical_trial, hydroxychloroquine, drug, study, remdesivir, hcq, tocilizumab, therapy	Clinical Trial
11	Cell, ace2, expression, receptor, sarscov2, tissue, human, virus, lung, pathway	Pathogenic Mechanism
12	Test, assay, testing, detection, sample, sarscov2, positive, specimen, sensitivity, antibody	Diagnosis
13	Child, symptom, pediatric, neurological, report, infection, infant, case, sarscov2, reported	Paediatrics
14	Social, crisis, health, economic, pandemic, impact, policy, consequence, psycinfo_database, right_reserved	Socio-economic Impact
15	Cancer, icu, intensive_care, treatment, ventilation, mechanical_ventilation, requiring, therapy, lung_cancer, ecmo	Oncology
16	Recommendation, risk, guideline, consensus, healthcare_worker, guidance, management, ibd, transplant, expert	Guidelines
17	Protein, sarscov2, compound, drug, target, vaccine, spike_protein, binding, inhibitor, epitope	Virus Structure
18	Virus, sequence, genome, human, bat, sarscov2, mutation, coronaviruses, genetic, animal	Genomic Sequence
19	Resident, person, county, older_adult, household, state, united_state, black, population, among	Demographics
20	Level, coagulation, thrombosis, ddimer, severe, elevated, crp, platelet, coagulopathy, aki	Haematology
21	Social_medium, information, public, video, news, medium, tweet, hand, misinformation, India	Communication
22	Use, vitamin, medication, drug, arb, angiotensin, angiotensin_receptor, ace_inhibitor, blocker, reninangiotensin_system	Existing Treatments
23	Liver, respiratory, skin, coinfection, ultrasound, gastrointestinal_symptom, diarrhea, imaging, symptom, fecal	Gastroenterology
24	Mask, aerosol, device, droplet, blood, particle, app, airborne, filter, mobile	Airborne transmission protection
25	Pregnant_woman, pregnancy, woman, maternal, pregnant, birth, neonatal, delivery, suspected, radiology_department	Pregnancy

*After optimising the LDA model, 25 topics were produced. The topics are listed in descending order of prevalence in the corpus. The top 10 words (middle column) are chosen based on relevance to the topic, with lambda = 0.6 (15). The topic summary (last column) is based on the consensus of the authors after reviewing the top 20 words and the top 10 abstracts*.

**Figure 3:**
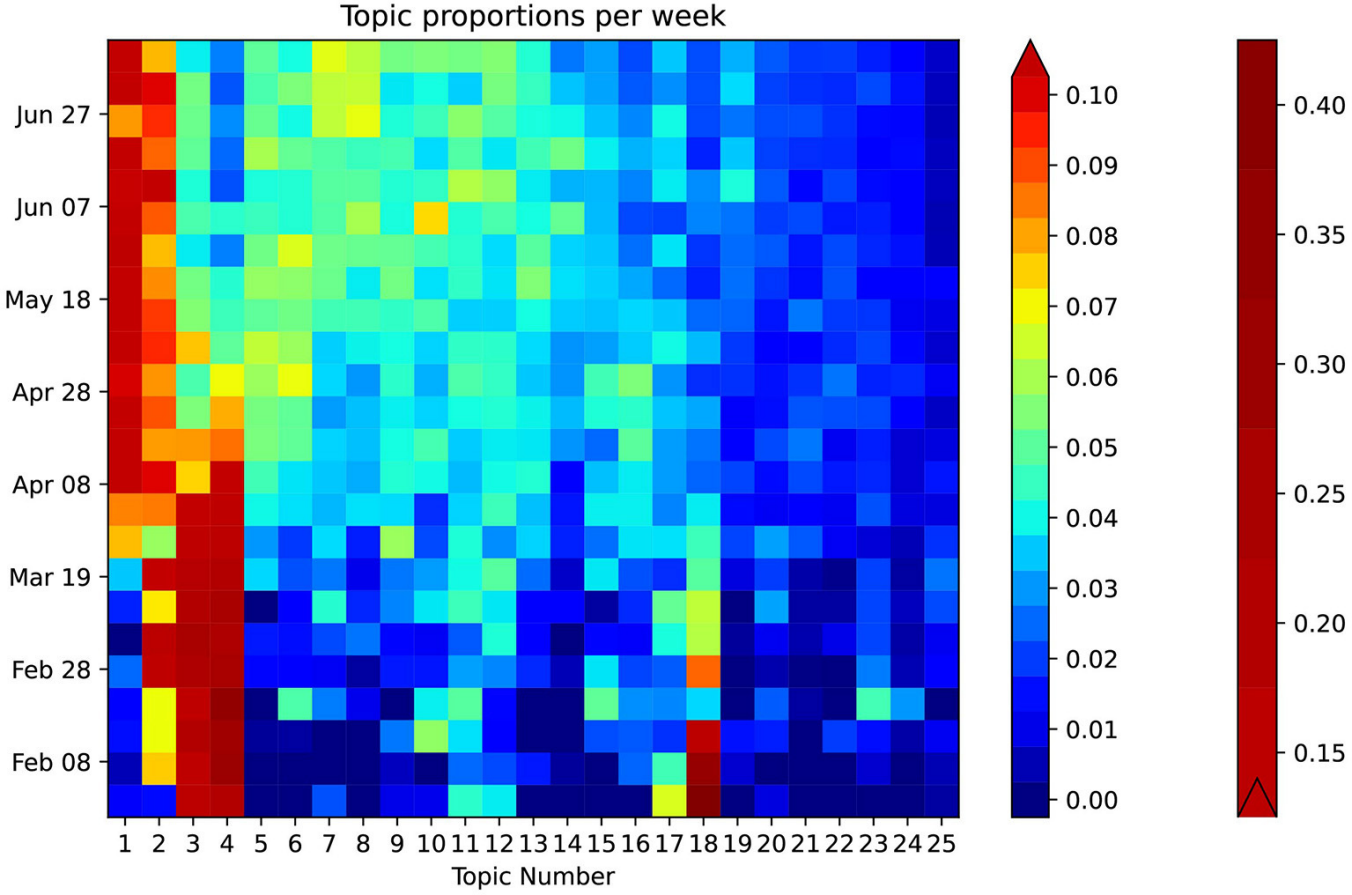
Heatmap showing weekly temporal topic trends in PubMed®. The heatmap is enhanced using a bilinear scale for an intuitive visual representation of the temporal trends of COVID-19 topics. The topics are sorted in descending order of proportion on the horizontal axis. Exemplary trends can be observed: Topic 1 increases around April, while, simultaneously, Topic 4 decreases. Certain topics such as 24 and 25 show a consistently low topic proportion throughout.

**Figure 4:**
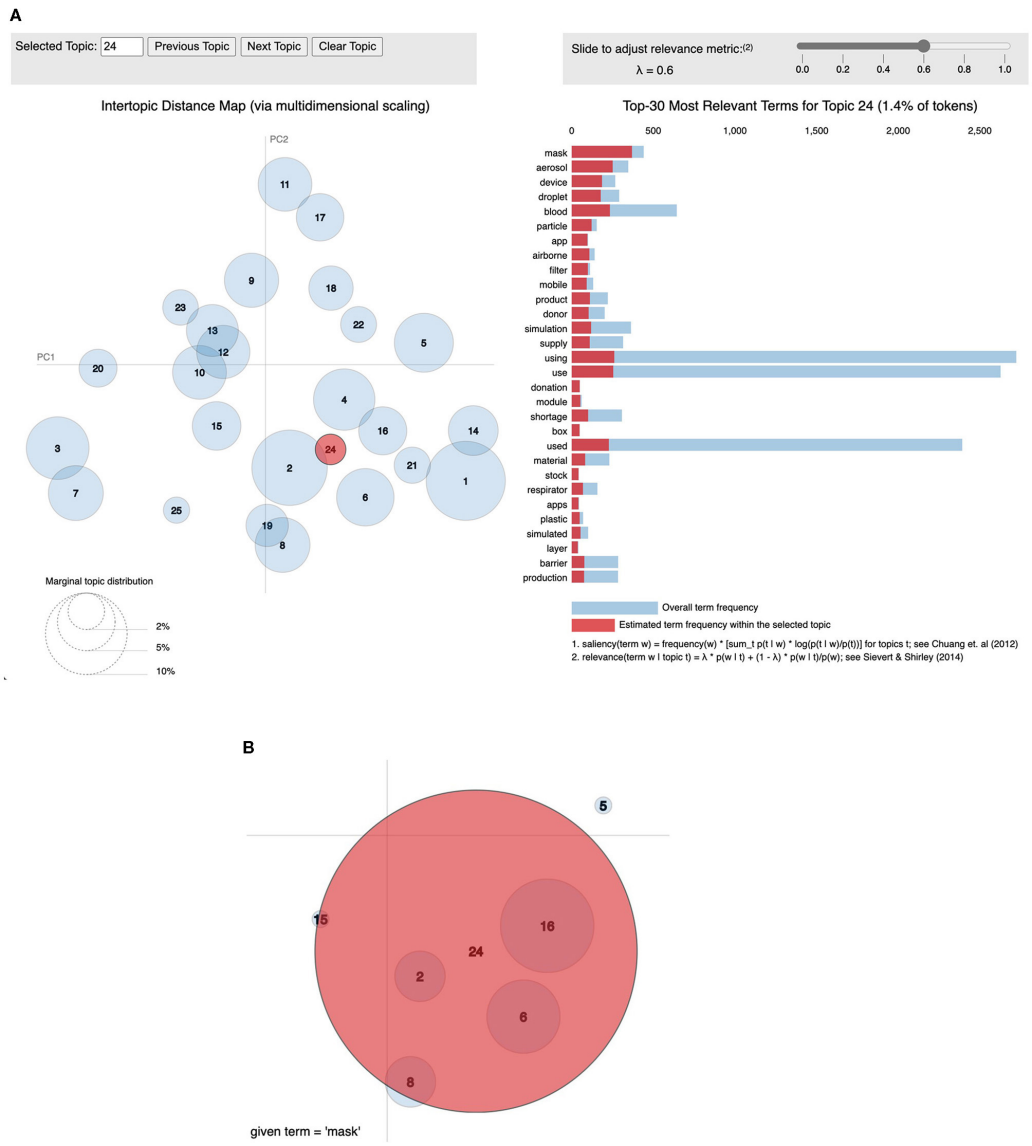
Visualization of topics using pyLDAvis. This is a visualisation produced using the pyLDAvis module (15). **(A)** The left shows an inter-topic distance map created using classical multidimensional scaling. The right shows the words in Topic 24 ordered by relevance set to 0.6 (15). **(B)** The word “mask” has the highest weight in Topic 24 (2.3%) when compared to others: Topic 16 (0.078%), Topic 6 (0.033%), Topic 2 (.0090%), Topic 8 (0.018%).

Figure 3 provides a general breakdown of how topic proportion changes over the timeframe. The weekly topic proportions for the PubMed® test data were all within the bounds of the training data and are plotted as the top three rows in the heatmap, which show hardly any visible discrepancies. Most topics start at some high/low proportion, then trend in the opposite direction and begin to level off in April 2020. Others follow a relatively steady change with time. The highest topic proportion of 41% is during January 2020 in Topic 18, “genomic sequence.” Topic 4, “disease outbreak,” has the highest mean weekly topic proportion of 13.5% (STD 10.5). Topic 24, “airborne transmission protection,” has the lowest mean of 0.98% (STD 0.66). This relatively lower topic proportion, as also illustrated in Figure 4A, was striking, because the usage of masks is a highly emphasized guideline (16). We performed a *post hoc* analysis by analysing the proportion of the term “mask” (as shown in Figure 4B) in different topics: Topic 24 (“airborne transmission protection”) 2.3%, Topic 16 (“guidelines”) 0.078%, Topic 6 (“surgery”) 0.033%, Topic 2 (“epidemiology”) 0.0090%, Topic 8 (“mental Health”) 0.018%.

**Figure 5:**
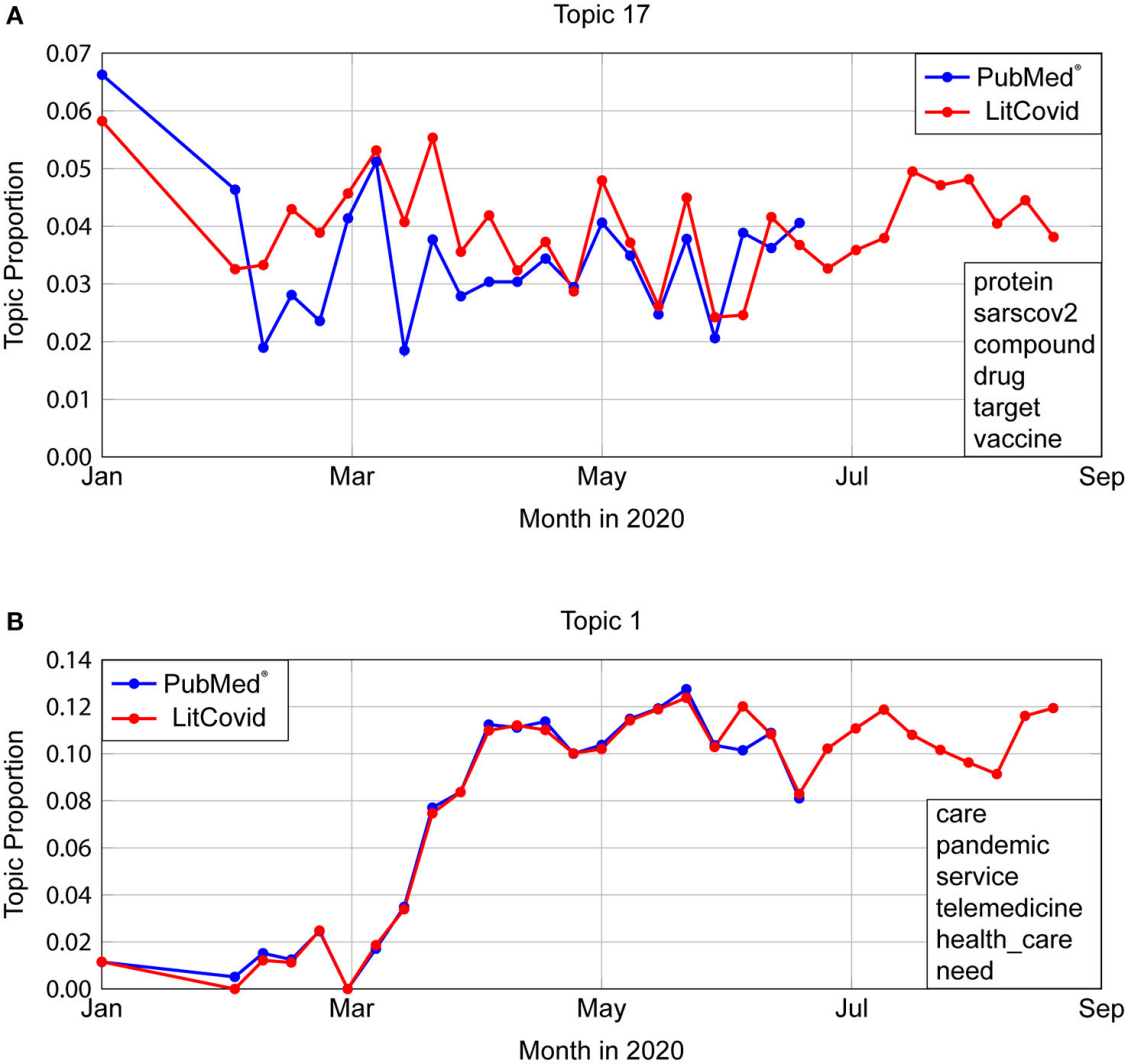
Comparison of temporal trends in PubMed® and LitCovid. **(A)** Topic 17 depicts the most dissimilar topic trends, with a Normalised Euclidean Distance (NED) of 0.0604. Even though LitCovid's dataset was also taken from PubMed®, the mild variation can be attributed to differences in search queries (described in Figure 2). **(B)** Topic 1 represents the most similar trends (NED = 0.0303).

Applying our LDA model to LitCovid abstracts shows that temporal topic proportions have a very strong agreement with an average Normalized Euclidean Distance (NED) of 0.0303 (STD 0.0128). The most dissimilar looking graph is shown in Figure 5A, which is Topic 17, “Virus Structure” with NED 0.0604. Even though LitCovid's dataset was also taken from PubMed®, the mild variation can be attributed to differences in search queries (as described in Figure 2). The most similar trends occur for Topic 1 (“Health care, telemedicine”) with NED = 0.0303, as show in Figure 5B.

**Figure 6:**
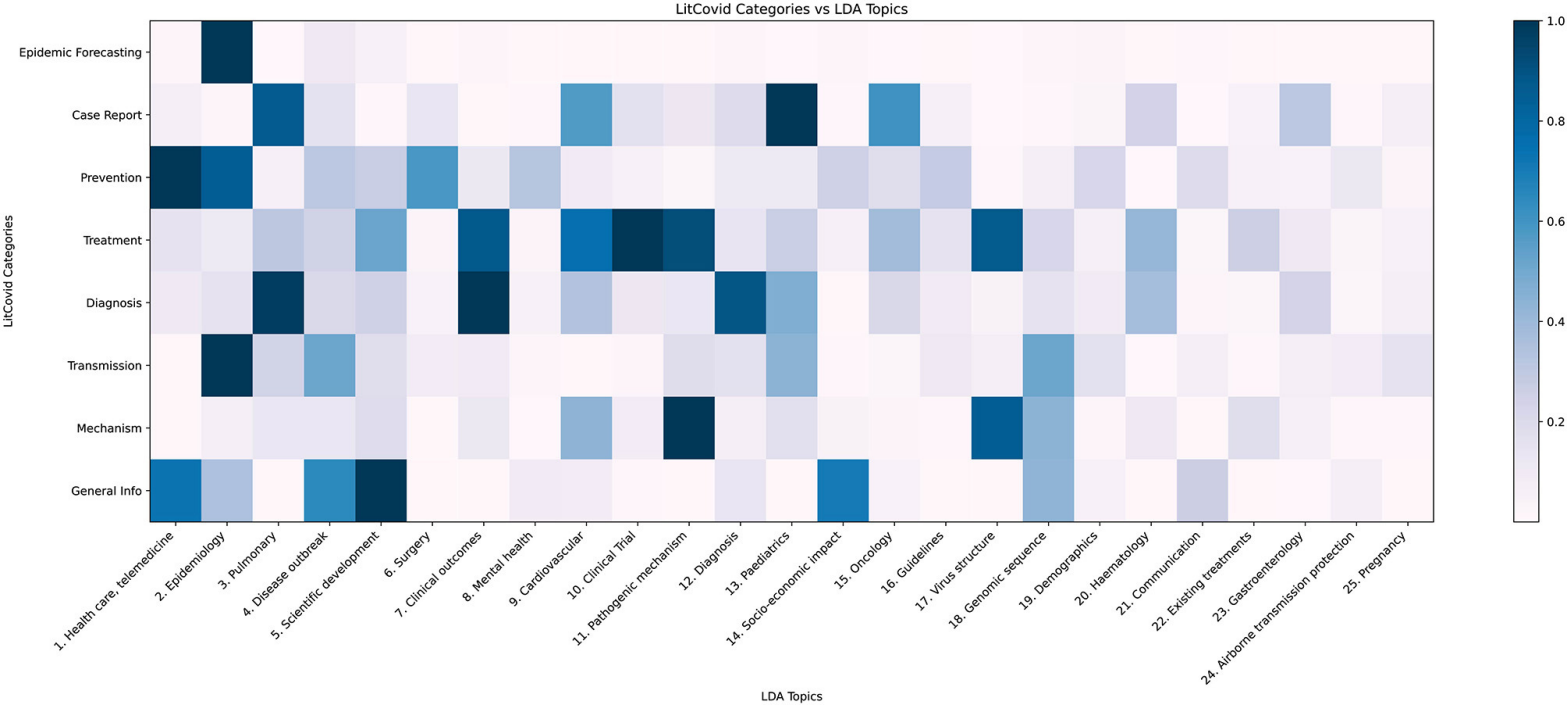
LDA topic distribution across LitCovid categories. LitCovid divides COVID-19 research into eight categories. Our LDA topic model, when applied to each category, returns a topic distribution, which is normalised (dividing by the largest proportion in the category) and plotted as the heatmap.

In Figure 6, applying our model to the LitCovid categories shows that the LitCovid category “Epidemic forecasting” is most similar to LDA Topic 2, “Epidemiology” as it has a topic proportion of 74%. The most intuitive category to subdivide is “General Info”; our topic model split it into “Scientific Development” (21%), “Health Care, Telemedicine” (16%), “Socio-economic Impact” (15%), “Disease Outbreak” (14%), “Genomic Sequence” (9%) and “Communication” (6%). Another notable subdivision is of the “Case Report” category into the relevant medical fields of “Paediatrics” (20%), “Pulmonary” (18%), “Oncology” (13%), and “Cardiovascular” (12%), as well as identifying the under-represented topics “Haematology” (5%) and “Gastroenterology” (6%).

**Figure 7:**
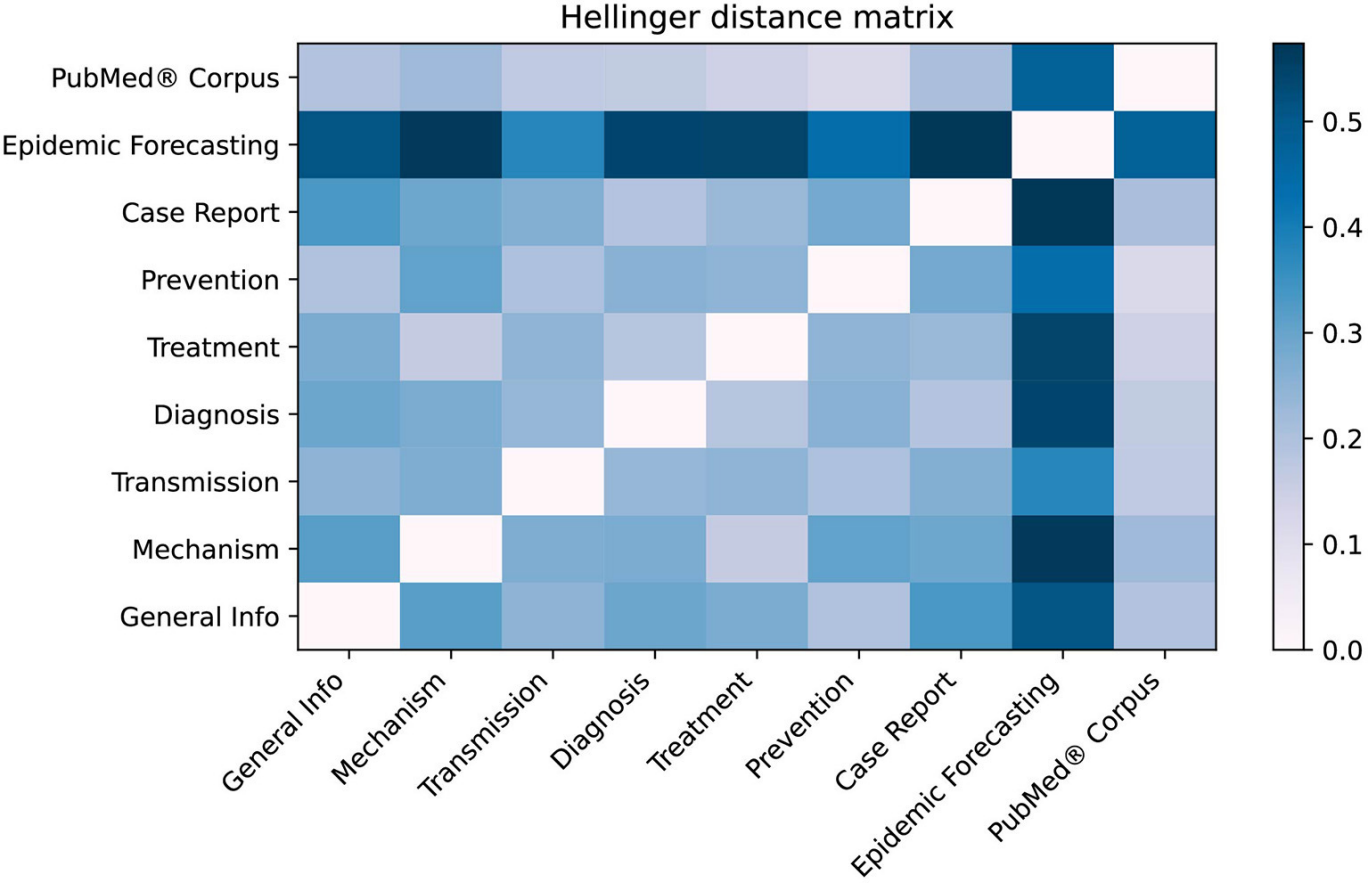
Hellinger distance matrix comparing LitCovid categories and PubMed®. The Hellinger distance, which is used to quantify the dissimilarity between two probability distributions, was used to compare LitCovid categories against each other as well as the PubMed® corpus. This distance was calculated using the distribution of the 25 topics shown in Figure 6. For example, the most dissimilar categories are “Epidemic forecasting” and “Case Report” (H = 0.572).

As shown in Figure 7, the LitCovid categories with the most overlap are “Treatment” and “Mechanism” with H = 0.160, whereas the least overlap is for “Epidemic forecasting” and “Case Report” with H = 0.572. When compared to the PubMed® corpus, “Prevention” has the lowest Hellinger distance of H = 0.116, suggesting that the “Prevention” category may be too broad. Conversely, “Epidemic Forecasting” has the greatest Hellinger distance (H = 0.476), suggesting that it is a well-defined category.

## Discussion

We provide a generalisable NLP methodology to extract abstracts from PubMed®, create an optimised LDA topic model, and visualise temporal trends. Applying our model to LitCovid abstracts helps to identify trending and under-represented research areas as well as subdivide its categories into relevant topics. We find that an important topic related to masks and PPE is under-represented in population-based research.

The optimised LDA model developed in this study identifies 25 topics with no significant overlap, and all relate to the COVID-19 pandemic. Our model is trained and tested on a much larger number of documents compared to previous studies, and we believe our topic interpretation is improved using NLP techniques such as the use of bi-grams and ordering terms by relevance rather than frequency (further details are in Supplementary Material). Therefore, our model is comprehensive, and our analysis is in-depth. Topics not previously identified include “Pathogenic Mechanism,” “Cardiovascular,” and “Demographics” (Topics 9, 11, and 19). Previous topic models developed using research publications provide broad categories and do not sufficiently characterise the social science aspect of COVID-19 (6, 7, 17, 18). In contrast, our model covers this field in the topics “Mental Health,” “Socio-economic Impact,” and “Communication” (Topics 8, 14, and 21, respectively), representing 10.2% of the PubMed® test corpus.

We developed a customised heatmap shown in Figure 3 representing topic trends on a holistic scale. This visualisation allows for intuitive understanding compared to the use of classic line graphs, which can be cumbersome to review, especially for a larger number of topics. From January to March 2020, Topic 18, “genomic sequence” has an unusually high topic proportion given its lower proportion in the overall corpus. This trend correlates well with our expectation as it was initially a hot topic in social media (19). The heatmap also clearly shows that Topic 24 (“Airborne Transmission Protection”) has a lack of adequate representation. As a direct application of our topic model, we zoomed into the word “mask” (Figure 4B), demonstrating its representation in very few topics, including Topic 24, 16 (“guidelines”), and 6 (“surgery”). Moreover, the relative weight of “mask” within Topic 16 is two orders of magnitude less than Topic 24. Considering the existing relationship between masks and the spread of COVID-19 (20), one would expect “mask” to have a greater representation in “Epidemiology.” The lack of research in this area suggests that a critical topic for future research is the usage of masks in a public context (21).

Analysis of temporal trends in Figure 3 includes both the training and test data from PubMed®, which does not show many discrepancies between the two, suggesting that both our model and methodology are applicable on other relevant datasets. Existing research papers (4–6) limit their analysis by focusing only on the corpus they trained on. However, we perform further testing and analysis on related articles outside of our training set. Applying our methodology to LitCovid shows that the temporal trends are largely the same, further validating our hypothesis. When they differ, such as in Figure 5A, the shape remains similar, with disagreeing values, which can be attributed to the difference in search queries when curating the datasets we analyse.

PubMed® is a central repository for biomedical research literature, containing search tools to identify publications based on search queries. It lacks comprehensive tools to analyse publications for topic identification. LitCovid uses a combination of human curation and machine learning to provide eight broad categories for COVID-19 research. By applying our NLP methodology, we find that many of these general categories can be subdivided into multiple relevant topics, which provides more comprehensive insights for future specialised research. For example, the inherently broad category of “General Info” is split into more specific topics including socio-economic impact, communication, and telemedicine. Another subdivision we believe to be useful is for the category of “case report”; our model split it into six medical fields, including paediatrics, cardiovascular, and gastroenterology.

Figure 7 shows there is measurable overlap between LitCovid's categories, some of which is to be expected because they all contain the overarching theme of COVID-19. Interestingly, our model shows that the most heterogeneous category, “Prevention,” comprises the topics “Health Care,” “Telemedicine,” “Epidemiology,” “Surgical Procedures,” and “Mental Health.” One would not expect some of these, but rather it would be more reasonable to have “Guidelines” as a core topic. The closest categories identified in Figure 7 are “Treatment” and “Mechanism,” which is reasonable because effective preventative treatment should be inhibiting a mechanism. Closer analysis using Figure 6 shows that “Mechanism” is almost a subset of “Treatment,” with the key difference being that “genomic sequence” is a strong topic in “Mechanism,” but not in “Treatment.” We believe that these categories can be ambiguous to a researcher, and our methodology would significantly improve online literature hubs through more specific subdivisions.

As we performed searches in LitCovid, we noticed that abstracts were sometimes not relevant to the query even though a relevance option is provided. Improved comparative relevance of a topic within a research paper can add value for researchers as it distils a large mass of research papers by the strength of their major topics. Future research can utilise our methodology to enhance online literature hubs by ordering documents based on the proportion of topics.

Although the LDA topic model can be updated with new research, further investigation on hyperparameter adjustment will be required to identify new topics. However, our methodology is simple to re-run and provide up-to-date trends. To evaluate the temporal trends, we use the date of publication, because we found that not all articles had the research date. Although the publication date does not accurately reflect the time when the research was performed, for our purpose, our temporal visualisation disseminates the trending topics from the under-represented ones, which we believe is more crucial to the researchers. Another potential limitation in our dataset is the exclusive use of English abstracts. Since LDA is essentially a clustering algorithm, if more than one language is used, the topic model will likely return duplicate topics in different languages, which is redundant for our purpose. Instead, since many research papers written in other languages provide abstracts translated into English (22), this further justifies our use of abstracts instead of papers. Even though a significant number of publications are not analysed due their lack of abstracts, we believe this would not affect our identification of topics, given that it is reasonable to assume that this is random and not biased against any topic.

NLP techniques applied with LDA topic modelling results in a comprehensive topic listing and identification of important temporal trends. Our methodology has the potential to complement existing literature hubs. We identify topics for further research, such as studies on masks that may be of significant public interest.

## Data Availability Statement

The original contributions presented in the study are included in the article/Supplementary Material, further inquiries can be directed to the corresponding author/s.

## Author Contributions

SA, AA, and AG: data generation. AA, SA, AG, and HG: data analysis and manuscript. AG, SA, AA, and HG: statistical analysis. HG: final manuscript approval. All authors contributed to the article and approved the submitted version.

## Conflict of Interest

The authors declare that the research was conducted in the absence of any commercial or financial relationships that could be construed as a potential conflict of interest.
