# Supplementary material for: Trends in COVID-19 Publications: Streamlining Research Using NLP and LDA
Source: Front Digit Health. 2021 Jul 6;3:686720. doi: 10.3389/fdgth.2021.686720 (PMC8522017; doi:10.3389/fdgth.2021.686720)
Supplement: Supplementary file 1 [file Data_Sheet_3.PDF]

Supplementary Materials

1. Overview of Latent Dirichlet Allocation

Latent Dirichlet Allocation is a "probabilistic model for collections of discrete data such as text corpora" [1]. Each word is modelled as a mixture of topics, and each topic is modelled as a mixture based on topic probabilities. The Gensim package in Python uses an unsupervised technique to learn the topic model.

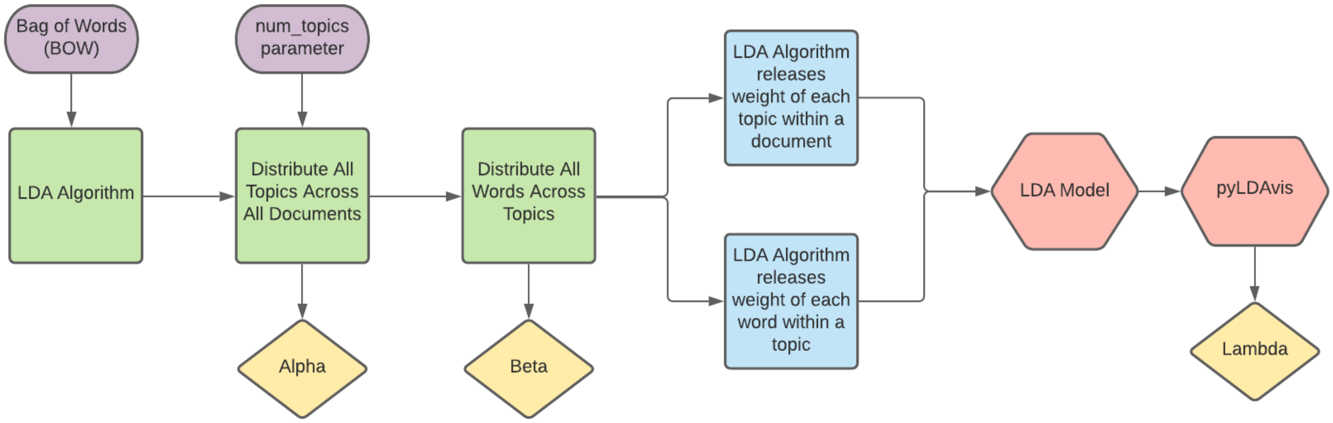

Fig 1: Latent Dirichlet Allocation (LDA) Algorithm
The process used by the LDA algorithm to determine the distribution of documents across the topics and the distribution of words across topics. Explanations of Alpha and Beta Values are described below in text.

The alpha value represents the distribution of the topics across the documents. A high alpha value indicates that the topics are less spread out across the documents, i.e. topics in a document are more equally weighted. Conversely, a low alpha value indicates that topics are more spread out across the documents, resulting in a few topics having a stronger weighting in documents [2].

The beta value represents the distribution of the words across the topics. A higher beta value causes more words to be concentrated in a single topic. As a result, each topic consists of a large number of significantly associated words. Conversely, a lower beta value causes the words in the corpus to be more spread out across all topics [2].

pyLDAvis is a visualization that depicts the distribution of the topics across the corpus as well as the distribution of words across topics. The lambda parameter in pyLDAvis helps determine the relative relevance of each term within a topic [3]. For each topic, we examined the top 10 abstracts and determined that the lambda=0.6 provided most consistent results.

2. Methodology

The data extraction and pre-processing steps for developing the Bag of Words (BOW) representation are outlined below.

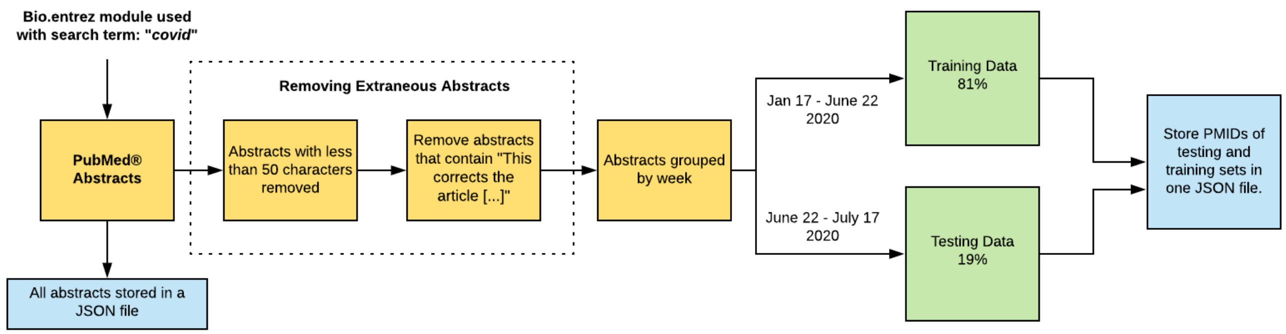

Fig 2: Extraction Process for PubMed® Dataset
This flow chart shows the extraction and storage of abstracts from PubMed® . The abstracts were stored in a JSON, whereas the PMIDS for testing and training were stored separately. A similar process was used for the abstracts in LitCovid.

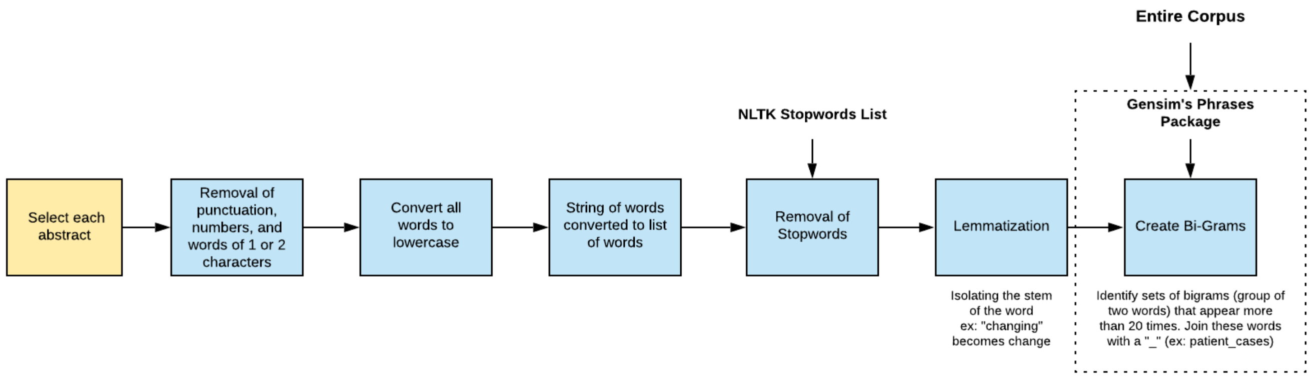

Fig 3: Pre-Processing Process of Datasets
Before transforming to BOW, all abstracts were pre-processed during run time (i.e. they weren't stored). However, the bi-grams model was stored for the training set, which was applied to all other datasets during pre-processing. This was to maintain consistency of the bi-grams.

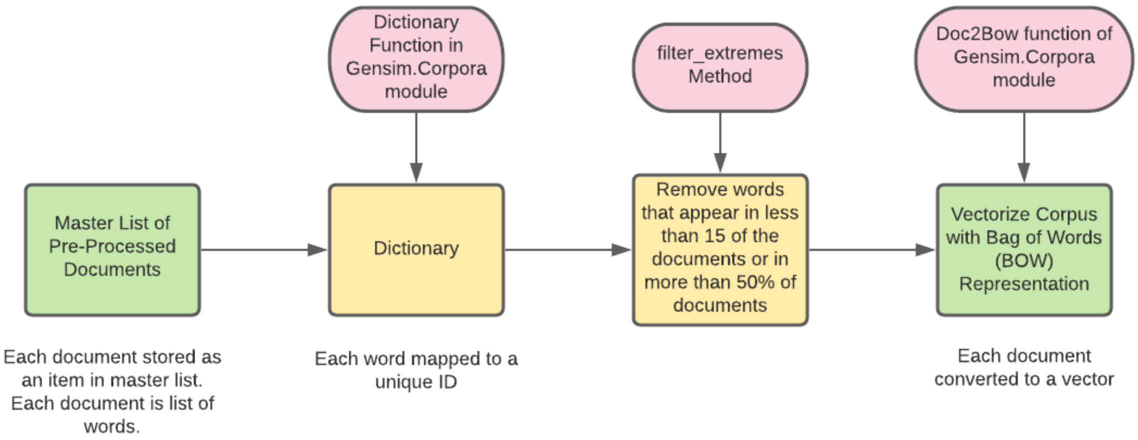

Fig 4: Developing a Bag of Words (BOW) Representation

The pre-processed abstracts were converted to a BOW representation. Each document is represented as a frequency distribution of word IDs. The mapping for word to ID is based off of the training set. This is used on all other datasets when converting to BOW.

3. Optimising the LDA model

All optimisations were done against the coherence value of the model. Coherence value is a measure of the coherence of the words identified within a topic. The more similar and related the words within a topic, the higher the coherence of the topic [4].

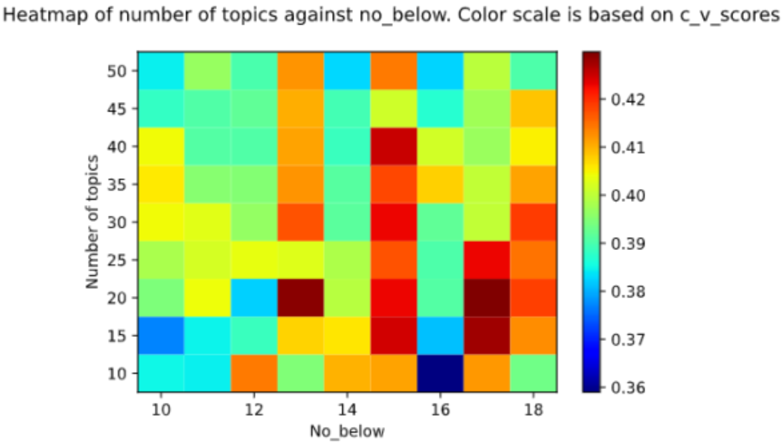

Fig 5: Optimising "No_Below" Value Based on Coherence Value Scores ("c_v_scores")
To determine our optimised no_below value (parameter of filter_extremes method), we developed the following heat map.

The no_below metric represents the minimum number of documents that a word must be found in for it to be included in our BOW representation. The maximum coherence value is achieved at a no-below value of 15. Therefore, we removed words that were found in less than 15 of the documents of the corpus.

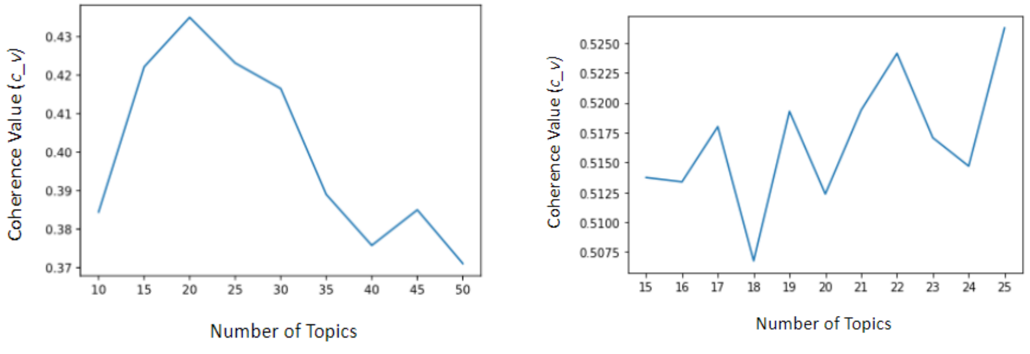

A **B**

Fig 6: Optimisation of Number of Topics Using Training Dataset
(A) Stepping by 5 topics in a range of 10-50 topics revealed that the coherence value is maximised for 15-25 topics.
(B) Stepping by 1 topic in a range of 15-25 topics revealed that coherence value is maximised for 25 topics.

**num_topics** (see Fig 6 for further detail)	25
**chunksize**<br>"Number of documents to be used in each training chunk" [5].	14000
**alpha**	Auto
**beta**	Auto
**passes**<br>"Number of passes through the corpus during training" [5].	40
**iterations**<br>"Maximum number of iterations through the corpus when inferring the topic distribution of a corpus" [5].	400
**random_state**	2020
**eval_every**<br>"Log perplexity is estimated every that many updates" [5].	None

Table 1: Final Parameters used to train LDA model
We have a random_state of 2020 (random seed) to ensure that the LDA model produces similar results every time we run the model.

4. Temporal Evolution of Topics

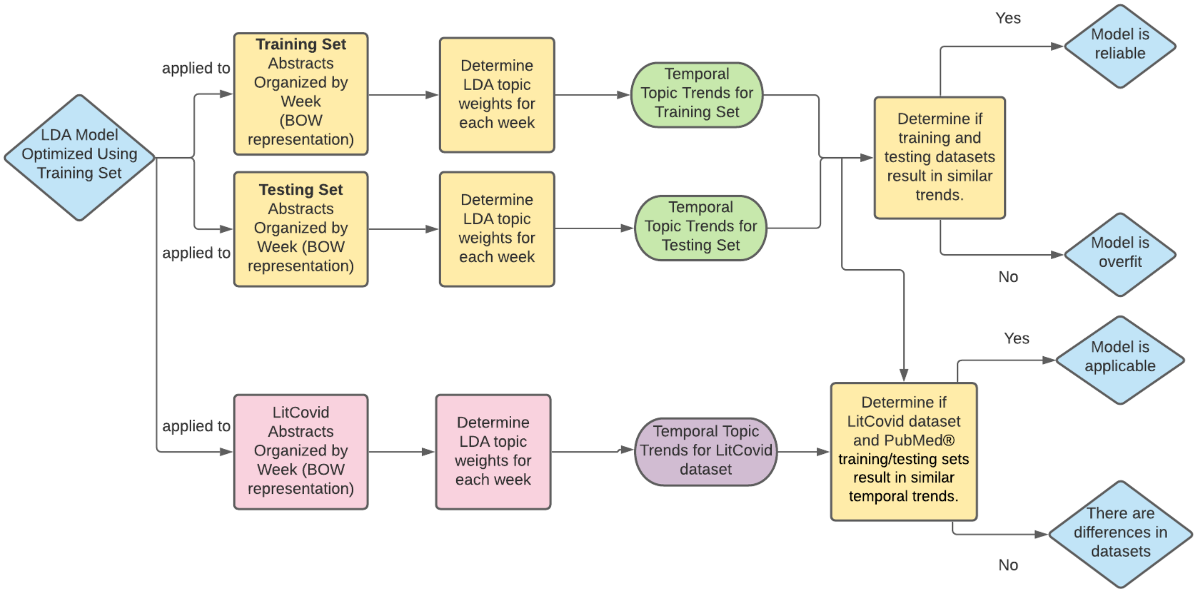

Fig 7: Developing Temporal Topic Trends
Temporal trends were first developed using PubMed® training and testing datasets. PubMed® temporal trends were compared against temporal trends produced using LitCovid abstracts. Overfitting indicates that the model is only applicable to the training set. Therefore, an overfit model would not be applicable to other datasets.

5. Topic-Category Comparison with LitCovid Dataset

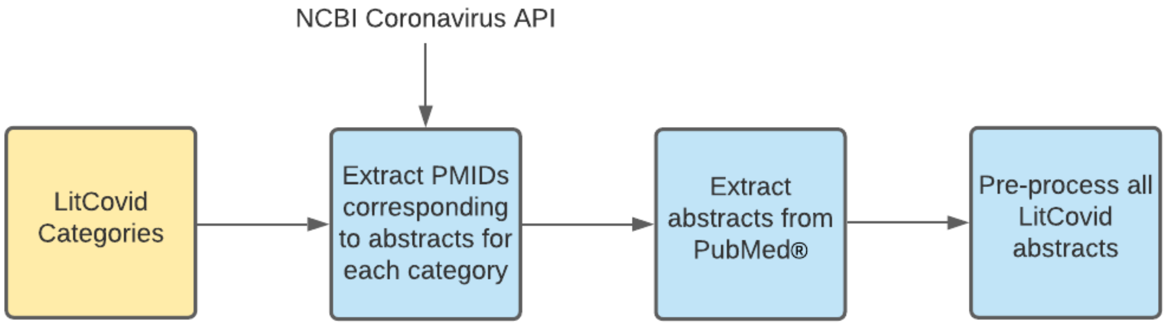

Fig 8: Extraction of Abstracts corresponding to categories in LitCovid
PMIDs were extracted from the NCBI Coronavirus API, which were then used to get the abstracts from PubMed. All of these abstracts were pre-processed in runtime.

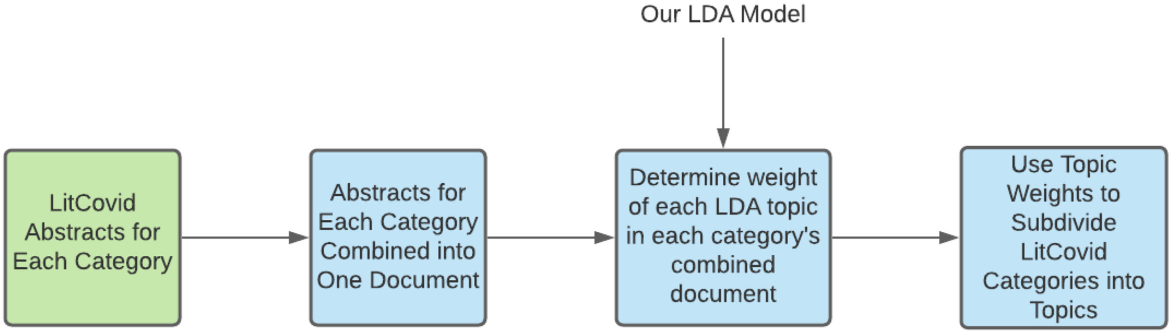

Fig 9: Subdivision of LitCovid Categories into LDA Topics
This process was used to create a heatmap illustrating the subdivision of the 8 LitCovid Categories into the corresponding weights of the 25 LDA Topics.

References:

[1] D. M. Blei, A. Y. Ng, and M. I. Jordan, "Latent Dirichlet Allocation," *J. Mach. Learn. Res.*, vol. 3, no. Jan, pp. 993–1022, 2003, Accessed: Oct. 03, 2020. [Online].

[2] "LDA Alpha and Beta Parameters - The Intuition." https://www.thoughtvector.io/blog/lda-alpha-and-beta-parameters-the-intuition/ (accessed Oct. 03, 2020).

[3] C. Sievert and K. Shirley, "LDAvis: A method for visualizing and interpreting topics," presented at the Proceedings of the Workshop on Interactive Language Learning, Visualization, and Interfaces, Baltimore, Maryland, USA, 2014, doi: 10.3115/v1/w14-3110.

[4] M. Röder, A. Both, and A. Hinneburg, "Exploring the Space of Topic Coherence Measures," *Proceedings of the Eighth ACM International Conference on Web Search and Data Mining - WSDM '15*. 2015, doi: 10.1145/2684822.2685324.

[5] P. S. Radim Rehurek, "Software Framework for Topic Modelling with Large Corpora," 2010, Accessed: Oct. 06, 2020. [Online]. Available: http://citeseerx.ist.psu.edu/viewdoc/summary?doi=10.1.1.695.4595.
