# Supplementary material for: Trends in COVID-19 Publications: Streamlining Research Using NLP and LDA
Source: Front Digit Health. 2021 Jul 6;3:686720. doi: 10.3389/fdgth.2021.686720 (PMC8522017; doi:10.3389/fdgth.2021.686720)
Supplement: Supplementary file 2 [file Data_Sheet_1.ZIP › FINAL_web_img/Interactive model for temporal trends.html]

Comparison of temporal trends for various topics in PubMed® and LitCovid

  

**Instructions for use:**
  
 Move the slider button in the x-axis to activate and view temporal trends for different topics and associated top five words (may need to scroll down)
