# Supplementary material for: Trends in COVID-19 Publications: Streamlining Research Using NLP and LDA
Source: Front Digit Health. 2021 Jul 6;3:686720. doi: 10.3389/fdgth.2021.686720 (PMC8522017; doi:10.3389/fdgth.2021.686720)
Supplement: Supplementary file 2 [file Data_Sheet_1.ZIP › FINAL_web_img/Interactive model for visualisation of topics.html]

Visualization of topics using pyLDAvis

  

**Instructions for use:**
  
- Lambda can be changed to values between 0 and 1. We set lambda to 0.6 (in
the top right)
  
- A topic can be highlighted by clicking on the topic number in the circles,
or using the "Next Topic" button on the top left
  
- After selecting a topic, one can mouse over various words to evaluate the
'weight' of a word for a particular topic (Example in Figure 4 of the
manuscript)

  
*Created using:
pyLDAvis*
